# Supplementary material for: Leadership and governance, financing, and coordination and their impact on the operationalization of health interventions in the humanitarian-development nexus in South Sudan
Source: PLoS One. 2025 May 23;20(5):e0312788. doi: 10.1371/journal.pone.0312788 (PMC12101634; doi:10.1371/journal.pone.0312788)
Supplement: S1 File — (ZIP) [file pone.0312788.s001.zip › Supporting Information/S2 Table.docx]

**S2 Table. Major Health Policies and Plans in South Sudan**

| **Document** |  | **Date(s)** |
| --- | --- | --- |
| National Health Policy |  | 2016-2026 |
| Reproductive Health Policy |  | 2013 |
| Family Planning Policy |  | 2013 |
| Health Sector Strategic Plan |  | 2023-2027 |
| Health Sector Development Plan |  | 2012-2016 |
| Reproductive, Maternal, Newborn, Child, Adolescent Health and Nutrition (RMNCAH and N) Strategic Plan |  | 2018-2022 |
