# Supplementary material for: Leadership and governance, financing, and coordination and their impact on the operationalization of health interventions in the humanitarian-development nexus in South Sudan
Source: PLoS One. 2025 May 23;20(5):e0312788. doi: 10.1371/journal.pone.0312788 (PMC12101634; doi:10.1371/journal.pone.0312788)
Supplement: S1 File — (ZIP) [file pone.0312788.s001.zip › Supporting Information/S1 Table.docx]

**S1 Table. Demographic Indicators in South Sudan, Regional (Sub-Saharan Africa), and Global Averages**

| **Indicator** | **South Sudan** | **Regional Average** | **Global Average** |
| --- | --- | --- | --- |
| Under 5 (U5) Mortality Rate (2021)  *(# of deaths per 1,000 live births)* | 99 | 73 | 38 |
| Neonatal Mortality Rate (2021)  *(# of deaths per 1,000 live births)* | 40 | 27 | 18 |
| Stillbirth Rates (2021)  *(# of deaths per 1,000 total births)* | 25.8 | 21.0 | 13.9 |
| Maternal Mortality Ratio (2020)  *(modeled estimate, deaths per 100,000 live births)* | 1,223 | 536 | 223 |
| Percentage of Girls Married by Age of 18 years (2022) | 52 | 31 | 19 |
| Fertility Rate, Total  (2021)  *(births per woman)* | 4.5 | 4.6 | 2.3 |
| Contraceptive Prevalence, *modern methods* (2023)  *(% of women ages 15-49)* | 6 | 50 | 46 |
| Literacy Rate, Adult Total (2018)  *(% of people ages 15+)* | 35 | 68  (2022) | 87  (2022) |
| Unemployment Rate, Total* (2022)  *(% of total labor force, ILO);*  **Does not account for informal economy* | 12.4 | 6.3 | 5.3 |
| Human Development Index (2022)  *(0 to 1)* | 0.381 | 0.549 | 0.739 |
| Life Expectancy; Male, Female (2023)  *(years)* | 55, 58 | 59, 63 | 71, 76 |
| Physicians per 10,000 population (2021) | 0.4 | 2.9  (Africa Region) | 16.3 |
| Nurses and Midwives per 10,000 population (2021) | 3.6 | 12.9  (Africa Region) | 39.4 |
| Prevalence of Severe Food Insecurity in the Population (%) | 63.2  (2021) | 24.6  (2019) | 10.5  (2019) |
