# Supplementary material for: Leadership and governance, financing, and coordination and their impact on the operationalization of health interventions in the humanitarian-development nexus in South Sudan
Source: PLoS One. 2025 May 23;20(5):e0312788. doi: 10.1371/journal.pone.0312788 (PMC12101634; doi:10.1371/journal.pone.0312788)
Supplement: S1 File — (ZIP) [file pone.0312788.s001.zip › Supporting Information/S4 Table.docx]

**S5 Table. Documents Reviewed Prior to On-Site Data Collection**

| **No.** | **Document** | **Source** | **Policy/Strategy** | **Key Areas of RMNCAH** | **Nexus-Specific Context (if applicable)** |
| --- | --- | --- | --- | --- | --- |
| 1 | South Sudan National Health Policy (2016-2026) | MoH South Sudan | ***Overview:*** Policy encompassing national health system and services offered through the MoH and relevant sub-departments  ***Relevant Content:*** Decentralized health system; Universal Health Coverage/basic package of health and nutrition services (BPHNS); parallel supply chain systems; a focus on how to enhance epidemics, emergencies and disaster preparedness and response capability; for International Health Regulation, disaster risk reduction, prompt response, and recovery as a means to mitigate impact of adverse public health events. | MCH (including IMCI, ICCM) and SRH (including STI management and GBV) | ***Health policy purpose:*** supports "the country’s resolve to transition from emergency health systems support to sustainable health systems development" (vii). Content developed by government and development actors only. ***Core pillars covered:*** Leadership & Governance; Coordination, Preparedness & Planning.  ***Broader principles covered:*** focus on quality, implementing "Quality of Care and Safety" measures in service delivery. |
| 2 | National Health Sector Development Plan (2012-2016) | MoH South Sudan | ***Overview:*** Comprehensive plan to reduce maternal and infant mortality rates in South Sudan and improving health of people of South Sudan.  ***Relevant Content:*** Detailed strategic plan including addressing critical shortage of human resources for health, service delivery plan, M&E indicators for assessing health outcomes, costing for infrastructure, pharmaceuticals (reliance on World Bank), and health information systems. Broken down by each level within the health system (state, county, Payam, Boma) | MCH, SRH | Frames partnership with private sector, international community as a valuable opportunity such that "support rendered in relief and humanitarian assistance can be transformed into development assistance for the mammoth task of building our country and society" (vii). Stakeholders acknowledged are primarily development actors. Documents challenges facing new Emergency Preparedness and Response Department (related to communicable disease) (p. 7). ***Core pillars covered:*** Leadership, Financing, Analysis & Planning, Information Sharing (M&E)  ***Broader principles covered:*** Quality of services/care, empowerment of communities, equity, and access for remote communities (e.g., weak referral pathways), sociocultural and socioeconomic barriers. |
| 3 | Reproductive Health Policy (2013) | MoH South Sudan | ***Overview:*** Policy focused on sexual and reproductive health, more broadly RMNCH services that have been absent or insufficient due to conflict. Emphasizes a multisectoral approach including other line ministries  ***Relevant Content:*** Approximately 86.9% of women delivering outside of health facilities in SS; Poor infrastructure of health systems at all levels with an "urban bias" attributed to inadequate and inequitable distribution of resources leaving rural communities at disadvantage; frequent stock outs/drug shortages; limited M&E systems; absence/limited quality benchmarks; poor admin/management at different levels within decentralized system; "Dominance of individual NGOs and other international agencies in the control of the formal health system even though their primary mandate still remains humanitarian and emergency in nature. | MCH, SRH | **Health system challenge:** NGO/INGO control of formal health system despite humanitarian/emergency mandate. Overall development focus. ***Core pillars covered:*** Leadership & Governance, Coordination, Finance, Planning, Info Sharing  ***Broader principles covered:*** Human rights-based approach; Equity (urban vs. rural bias), socioeconomic barriers, multilingual/multiethnic barriers limiting effective reach of different populations; Gender Sensitivity, localization. |
| 4 | Reproductive Health Strategic Plan (2013-2016) | MoH South Sudan | ***Overview:*** Reformed community-engaged health system, with an associated structure of committees (governance), CHW teams (staffing), health offices (technical supervision), and home health promoters (support, referral).  ***Relevant Content:*** Includes specific targets for multiple areas of focus, including: reduction of maternal and infant morbidity and mortality rates through targeted services (e.g., ANC, PNC, comprehensive RH packages), GBV rates, health worker capacity and retention rates. Addresses other proxy indicators that impact poor health outcomes including high illiteracy rates, unemployment, and poverty. Create "enabling environment" for RH services, references WHO health system building blocks. | MCH, SRH, GBV | N/A; development-focused  ***Core pillars covered:*** Leadership & Governance, Coordination, Finance, Planning, Info Sharing  ***Broader principles covered:*** focus on special groups (e.g., youth), gender-sensitive lens, rights-based approach. |
| 5 | Family Planning Policy (2013) | MoH South Sudan | ***Overview:*** Policy encompassing family planning/birth spacing that is aligned with RH strategic plan, RH commodity security strategy, and health sector development plan and is under the Department of RH (MoH).  ***Relevant Content:*** Addresses MDGs 4, 5, 6 through family planning/birth spacing; Draws out supply/demand framework for FP. Combination of service delivery at both facility and community levels and access to information. Integration with other services/referral pathways (e.g., safe motherhood programs, GBV and human-rights programs, addressing HIV/STIs). Creating enabling environment (legal framework) for public and private sector involved with FP. Incorporating FP related indicators in DHIS/surveillance. Highlights service delivery (packages/elements) at all/each level(s) within the health system. | FP and integration with other relevant services. | States new political climate has "allowed the transition of the health system from a humanitarian and relief-based approach to a development approach" (p. 3) Otherwise, development-focused; involves development actors. ***Core pillars covered:*** Leadership & Governance, Analysis & Planning, Information Sharing  ***Broader principles covered:*** SRHR/human rights, enabling legal environment, youth-focused, quality of services |
| 6 | The Community Health System in South Sudan: The Boma Health Initiative (2016) | MoH South Sudan | ***Overview:*** Reformed community-engaged health system, with an associated structure of committees (governance), CHW teams (staffing), health offices (technical supervision), and home health promoters (support, referral).  ***Relevant Content:*** "Integrated package of health promotion, disease prevention. Maternal Child Health Programme Area: Immunization, newborn care, safe motherhood, men's reproductive health, STIs, ARH, maternal death surveillance, OB/GYN referral, SGBV prevention. Other relevant: Health education on breastfeeding, maternal/child nutrition, HIV/AIDS prevention, campaigns, and outreach for health promotion." | Infant/maternal mortality, birth registration | Contributions/partners include humanitarian and development actors. Program components include emergency preparedness for communicable disease outbreaks.  ***Core pillars covered:***  Leadership & Governance; Coordination; Finance; Preparedness & Planning; Information Sharing.  ***Broader principles covered:*** Norms, establishing standards and structure for CHW health initiative. |
| 7 | USAID Deliver Project, Final Country Report South Sudan (2012-2016) | USAID | ***Overview:*** Program/partnership between USAID, South Sudan MoH, and Essential Medicines Fund (EMF) to ensure medicines and commodities were procured, stored, and delivered to county level health departments and hospitals. Resulted from end of WB multi-trust fund. ***Relevant Content:*** Investment in commodity support and technical assistance—one major component was FP commodities; Support with supply chain management, storage, staff capacity. Project conducted a RH quantification and reproductive health commodity security (RHCS) assessment. Based on the assessment, a draft RHCS strategy was developed. | FP (leading to RH commodity security assessment) | Project by development partners involved delivery of medical commodities to prevent anticipated stockouts after the "end of a World Bank-managed, multi-donor trust fund activity" (p. 9); followed up with technical assistance (strengthening logistics system performance, increasing national commitment to commodity security, building sustainable capacity). Anticipates government will need donor support to maintain supplies for the foreseeable future. ***Core pillars covered:*** Preparedness & Planning, Leadership & Governance, Coordination, Information Sharing.  ***Broader principles covered:*** Access, Quality |
| 8 | Child Spacing & Family Planning in South Sudan: Knowledge, Attitudes, Practices and Unmet Need (2011) | Pillsbury et al. | ***Overview:*** Study to understand KAP and low acceptance rate of modern contraceptives/family planning in South Sudan. ***Relevant Content:*** Modern contraception in South Sudan is one of lowest rates globally. Study aims to understand KAP to help facilitate design of services that respond to needs and preferences of the South Sudanese people. Recommendations include destigmatizing/demystifying term of FP and preference as "birth or child spacing;" emphasize health benefits of birth spacing; engage husbands/partners; utilize midwives; address barriers for remote/HTR communities; health as an entry point, but limited impact of addressing GBV via FP services since there is low uptake of FP. | FP | Development-focused  ***Core pillars covered:*** Preparedness & Planning, Information Sharing, Leadership & Governance.  ***Broader principles covered:*** Sociocultural/religious norms (e.g., postpartum sexual abstinence, polygamy, unmarried pregnant adolescents), urban vs. rural dynamics. |
| 9 | Community Health Systems Catalog Country Profile: South Sudan (2016) | USAID, JSI, FHI360 | ***Overview:*** Assessment that builds on previous landscape assessments of community health systems globally, with a focus on South Sudan. ***Relevant Content:*** Provides an overview of 136 different interventions provided at the community level in South Sudan. Includes statistics on number of CHWs (or other community-level cadre), ratio of CHWs to beneficiaries, and types of services/interventions/supplies offered by "topic" (e.g., FP, MCH, Malaria, etc.) and focuses on human resources for health (HRH), health supplies, and service delivery broken down by: clinical services, health education, and community mobilization. | MCH, SRH | Community-level health committees engage in emergency preparedness activities. Otherwise, development focused.  ***Core pillars covered:*** Leadership & Governance, Information Sharing, Preparedness & Planning (health supplies).  ***Broader principles covered:*** health equity, right to health. |
| 10 | USAID Strategic Framework South Sudan (2020 to 2024) | USAID | ***Overview:*** Broad strategic framework for South Sudan with a focus on Resilience; 4-year scenario-based strategy for community-focused work to move past need for humanitarian aid. ***Relevant Content:*** Outlines a series of development objectives: development objective 1 (DO1): humanitarian assistance supports development, and development objective 2 (DO2): household resilience increased in targeted areas, and result 2.3 related to improved health outcomes, specifically family planning and MCH. These objectives include leveraging humanitarian assistance to build resilience, working toward convergence (different actors all working toward development objectives), enhancing planning scenarios since South Sudan is a shifting/dynamic landscape. USAID's J2SR: Journey to Self-Reliance (financing). "Supporting health service delivery via the Health Pooled Fund, Working against HIV/AIDS Provide access to immunizations, commodities, FP and SGBV services, Referral to other services. | MCH, FP, GBV, FSN, immunizations | Notes US government gave $5.6bn in humanitarian aid, $1.8bn for development; notes ongoing dependence on humanitarian assistance for survival and anticipates increased need in coming years. Strategic purpose: lay foundation for self-reliance. Specifies development hypothesis/theory of change to this end. Relevant themes: Leveraging humanitarian aid to build resilience; Engaging private sector for sustainable development; Donor coordination to boost humanitarian-development coordination. ***Core pillars covered:*** Preparedness & Planning, Information Sharing, Coordination, Financing.  ***Broader principles covered:*** Special groups, youth, gender, social cohesion. |
| 11 | Integrated Country Strategy, South Sudan (2022) | U.S. State Department | ***Overview:*** broad strategy from U.S. foreign policy standpoint for South Sudan in terms of HDP.  ***Relevant Content:*** Aligns with some aspects of USAID strategic Framework: Mission Goal 4: South Sudan improves its resilience to environmental, conflict driven, and economic shocks while decreasing its dependence on humanitarian assistance. (Incorporates USAID DO 1 and 2), Mission Objective 4.1: South Sudan extends equitable access to all communities to government-provided essential services that improve food security through agricultural production, natural resource management, health, education, and nutrition. | Health, non-specific | Reducing need of humanitarian assistance and promoting self-reliance as key goals; intent to use development tools to do so. Achieving infrastructural and political development objectives is understood to reduce humanitarian aid dependence. Intent to continue providing lifesaving emergency response services; crisis management understood to prevent further loss and return to civil war. Aims to shift burden of emergency response from international actors to S. Sudanese government.  ***Core pillars covered:*** Leadership & Governance, Preparedness & Planning. ***Broader principles covered***: human rights, gender equity, racial equity. |
| 12 | South Sudan Humanitarian Response Plan (2022) | OCHA | ***Overview:*** Plan based on humanitarian needs, health sector plan linked to resilience and health system recovery. ***Relevant Content:*** Health sector response focus on: RH, MCAH, GBV-related services, MH, disabilities, epidemic-prone areas through surveillance and detection (IDSR and EWARS), Ebola (EVD) preparedness and response; vaccinations; and referral pathways to non-health services. | MCH, SRH | Reports that UN Country Team analysis identified challenges to sustainable development and long-term needs; response intends to align with this " across the humanitarian-development-peace pillars" (p. 14). Describes role of United Nations Sustainable Development Cooperation Framework’s (UNSDCF) role in-country. Intended outcomes include the following, to "contribute towards strengthening the nexus between humanitarian response, resilience and development programming and peacebuilding (conflict sensitivity) " (p. 22): increasing HH resilience; integrate PSEA across sectors/clusters/working groups; health cluster collaboration with development actors to strengthen health system recovery, coping. Intention to engage development actors. ***Core pillars covered:*** Coordination, Leadership & Governance, Preparedness & Planning, Financing, Information Management.  ***Broader principles covered:*** quality of care, access, equity. |
| 13 | South Sudan Humanitarian Needs Overview (2022) | OCHA | ***Overview:*** Broad overview of humanitarian needs across the country. ***Relevant Content:*** One of poorest health systems (along with poor health infrastructure) globally; highest rates of maternal and infant mortality; low immunization rates; limited access to health services for most of the population (80% cannot access HFs in 1 hours’ time), Only 8% of HFs offer full BPHNS and only 53% of HFs are moderately functional; climatic shocks including flooding and droughts; high rates of GBV due to social norms and presence of armed groups; teenage pregnancy 30% for 15-19 year olds; 6% contraceptive prevalence rate. | MCH, FP, vaccines | N/A; emergency response focus.  ***Core pillars covered:*** Preparedness & Planning, Coordination, Leadership & Governance.  ***Broader principles covered:*** gender equity, human rights, IDP rights. |
| 14 | South Sudan Humanitarian Situation Report (September 2022) | WHO | ***Overview:*** General Situation Updated.  ***Relevant Content:*** Response to cholera, measles, and hepatitis E; preparedness for Ebola; emergency flooding, funding shortages. | Child Health, vaccinations | Donors include some development actors.  ***Core pillars covered:*** Preparedness & Planning, Coordination, Financing. |
| 15 | South Sudan Social Norms Assessment (2022) | MOMENTUM | ***Overview:*** Assessment of FP/RH social norms, menstrual hygiene, and management, GBV, health-seeking behavior; inform USAID/S Sudan social and behavior change efforts.  ***Relevant Content:*** Assessed via cross-sectional descriptive assessment (qual), KIIs, IDIs; Components considered: awareness, intentions, and decisions on contraceptive use; women's/girls' autonomy and agency; strategies, consequences; norms and social significance of menstruation, hygiene practices; expectations around sex before/outside marriage; GBV as reproductive coercion. | RH/FP, GBV | Note population's longstanding reliance on humanitarian assistance, level of ongoing need. Some humanitarian agencies provide dignity kits through schools; uncertainty as to consistency/duration of support.  ***Core pillars covered:*** Analysis & Planning, Information Sharing. ***Broader principles covered:*** Norms (in the sense of social and cultural injunctions). |
| 16 | Report: Fragility, Crisis Sensitivity, and Complexity (F2C) Assessment South Sudan (2022) | MOMENTUM | ***Overview:*** Needs assessment of SRH/MNCH+N services. ***Relevant Content:*** "Describes conflict- and stressor-related disruptions to various services: Skilled reproductive health care; Family planning methods and access to commodities; Training institutions for midwives; Private health facilities." | FP and implications for GBV, retaliation against healthcare workers | General but explicit concern for humanitarian-development nexus. Notes longevity and complexity of crisis state; attributes lack of country-wide sustainable development. Gives historical context for political instability and ongoing conflict. Describes how conflict and crisis interfere with access to MNCH services. Involvement/leadership of development actors on: GBV/early marriage; health service provision during disease outbreaks.  ***Core pillars covered:*** Analysis & Planning, Information Sharing. ***Broader principles covered:*** Humanitarian Principles (effects of conflict and shock on SRH/MNCHN services, advocacy to address issues related to system strengthening, protection, etc.). |
| 17 | Measuring health system resilience in a highly fragile nation during protracted conflict: South Sudan (2011–2015) | Odhiambo et al. | ***Overview:*** Examines health system resilience (HSR) in South Sudan. ***Relevant Content:*** Article examines 3 current HSR definitions: maintaining function, improving function, and achieving health system targets using data from South Sudan. 14 MNCH indicators from household surveys were used in 2011 and again in 2015 (2 years after protracted conflict) to construct a resilience index (RI). Measured relationship between resilience and stress level. | MCH, FP (including contraceptive prevalence) | Analysis of MNCH indicators explicitly seeks to measure health system resilience in a fragile context.  ***Core pillars covered:*** Preparedness & Planning, Leadership & Governance, Financing.  ***Broader principles covered:*** urban/rural disparities, access. |
| 18 | South Sudan: Program Year 2 Implementation Plan (2021) | MOMENTUM | ***Overview:*** 2nd-year programming to support health facility programs - focus on quality, mentorship, scaling, coordination, facility development.  ***Relevant Content:*** Increasing access/quality of integrated care in public and private sectors; increasing demand and utilization; enhancing resilience and inclusiveness of health system. | ANC, PNC, deliveries, IMNCI, immunization, child nutrition, FP, counseling/testing, PMTCT, GBV | Situates countries of focus on "humanitarian-development continuum." Key activities included EWARS assessment. Documents issues of hostility, violence toward NGO and humanitarian workers as impediments to accessing SRH care. Intent to develop complementary training materials. Intent to interface with humanitarian partners to "layer, sequence, and integrate risk scenarios, as well as other service delivery points of intersection to expand access and maintain continuity of quality services and interventions" (p.17).  ***Core pillars covered:*** Leadership & Governance; Coordination; Finance; Analysis & Planning; Information Sharing.  ***Broader principles covered:*** Quality (much discussion of M&E, including performance monitoring plan, MEL plan, learning agenda, data management); Norms (in reference to existing national frameworks). |
| 19 | South Sudan Health Resilience Assessment Report (2021) | MOMENTUM | ***Overview:*** Assessment of health system challenges in FP/RH/MNCH. ***Relevant Content:*** "Assessed via: secondary review of health system and service delivery, KIIs, FGDs, workshops. Components considered: community, primary, secondary, tertiary-level healthcare; health staff distribution, training, retention; HMIS; financing and resources; health service delivery (and equitable access); equipment and supplies; quality assurance; system governance and leadership." | FP, ANC, EmONC (basic and comprehensive), child immunizations | Notes humanitarian issues of ongoing concern that directly affect health system resilience. Often cites humanitarian needs overview to give context, describe key issues at hand. Notes health service delivery is both led and governed by international humanitarian and development entities, with a complicated service delivery system.  ***Core pillars covered:*** Leadership & Governance; Coordination; Finance; Preparedness & Planning; Information Sharing. ***Broader principles covered:*** Primarily focused on Quality and associated components, indicators, and challenges; Humanitarian Principles in its detailed review of shocks and stresses and anticipating risk scenarios. |
| 20 | South Sudan MIHR Quarterly Performance Report: Program Year 2, Quarter 2 (2022) | MOMENTUM | ***Overview:*** Support improved FP/RH/MNCH outcomes and strengthen the USAID/South Sudan resilience platform. ***Relevant Content:*** "Increasing access/quality of integrated care in public and private sectors; increasing demand and utilization; enhancing resilience and inclusiveness of health system. Service provision: commodities, equipment, reference materials, onsite mentorship, and medical and infection prevention and control supplies, expansion of services, coordination, social and behavior change pilot package; Trainings: quality of care, identifying shocks and developing interventions, ToT on emergence preparedness response." | MCH. SRH, FP, integrated service delivery | Notes significance of nexus to contexts of interest. Humanitarian and development actors/funders involved. Key objectives include ensuring MNCH access during crises, including health worker ToTs on emergency preparedness. ***Core pillars covered:*** Leadership & Governance; Coordination; Finance; Analysis & Planning; Information Sharing.  ***Broader principles covered: Quality***, Localization (in the sense of providing trainings, ToTs, coordinating with public as well as private sectors). |
| 21 | PY1 Program Overview South Sudan (2019/2020) | MOMENTUM | ***Overview:*** Support improved FP/RH/MNCH outcomes and strengthen the USAID/South Sudan resilience platform. ***Relevant Content:*** Technical leadership, quality service delivery, social and behavior change, capacity building, resilience, monitoring/eval/research/learning. | MCH, FP | Notes background context of ongoing humanitarian crisis. Donors and partners from both humanitarian and development entities. Key areas include resilience and "shock-responsive programming." Resilience activities include: "Develop a framework for layering, sequencing and integrating humanitarian & development assistance for MNCH and FP activities" (15).  ***Core pillars covered:*** Leadership & Governance; Coordination; Finance; Preparedness & Planning; Information Sharing. ***Broader principles covered:*** Primarily focused on assuring Quality (of services and integration); Localization (in the sense of training public and private service providers). |
| 22 | South Sudan Quarterly Report (August-September 2020) | MOMENTUM | ***Overview:*** Focus on transitioning the existing E2A project (implemented by IntraHealth) to MIHR by the end of December 2020. ***Relevant Content:*** " Transition activities: operations, social norms study, CHWs, dedicated health providers; FP service transition in 4 key health facilities; Program management startup, including security assessment; Procuring larger office space for MIHR team and hiring for new roles." | FP, SBC | Notes significance of nexus to contexts of interest. Attends to ongoing risk in activity planning.  ***Core pillars covered:*** Leadership & Governance; Coordination; Preparedness & Planning; Finance.  ***Broader principles covered:*** Primarily focused on Quality (maintaining services through transition). |
