# Supplementary figures and images for: Leadership and governance, financing, and coordination and their impact on the operationalization of health interventions in the humanitarian-development nexus in South Sudan
Source: PLoS One. 2025 May 23;20(5):e0312788. doi: 10.1371/journal.pone.0312788 (PMC12101634; doi:10.1371/journal.pone.0312788)

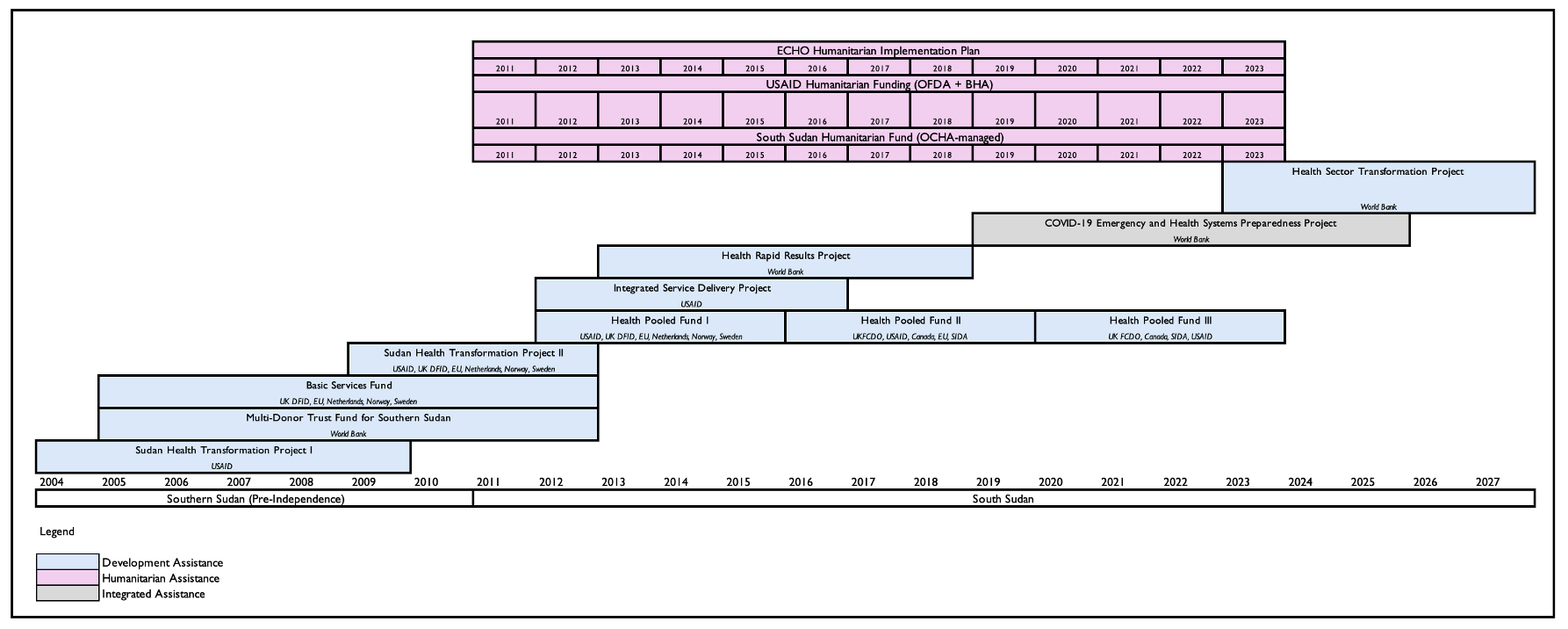

Supplement: S1 File — (ZIP) [file pone.0312788.s001.zip › Supporting Information/S3 Fig_Timeline of Major Humanitarian and Development Funds in South Sudan.tif]
