# Supplementary material for: Leadership and governance, financing, and coordination and their impact on the operationalization of health interventions in the humanitarian-development nexus in South Sudan
Source: PLoS One. 2025 May 23;20(5):e0312788. doi: 10.1371/journal.pone.0312788 (PMC12101634; doi:10.1371/journal.pone.0312788)
Supplement: Attachment — Submitted filename: Response to Reviewers_HDN South Sudan.docx [file pone.0312788.s003.docx]

**Response to Reviewers**

*PLOS ONE*

***Leadership and Governance, Financing, and Coordination and their Impact on the Operationalization of Health Interventions in the Humanitarian-Development Nexus in South Sudan***

____________________________________________________________________________________

Firstly, we would like to say thank you for the thoughtful reviews from the reviewers. They have been very helpful in improving our manuscript, and we are happy to submit a revised version.

Please find our response to the reviewers’ comments herein. Please note that the reviewers’ initial comments below are numbered and in black text and our responses are in red text.

____________________________________________________________________________________

**REVIEWER #1:**

This study is relevant to the context of South Sudan because of the continuous lack of progress in achieving health targets despite huge investments. The paper is however more of a project report than a scientific paper that links findings to the literature. To improve the manuscript, the following are suggested:

1. Improve the introduction section with more literature review that defines the issues around the health-development-peace nexus. This will help provide a context to make more informed inferences from your findings in the discussions.

The reviewer’s comment is noted and appreciated. To further orient the reader, additional background on the humanitarian-development nexus (HDN) from the literature has been incorporated into the introduction. In addition, a table has been added for some key distinctions between humanitarian and development assistance including the time-frame of assistance for each, coordination mechanisms, etc. These additions can be viewed in lines 82-133. Please note we refer to the “humanitarian-development nexus” (i.e., the HDN or the nexus) in this paper and not the “health-development-peace nexus” as mentioned by the reviewer. Please see response to reviewer 1, comment 7 on the peace component.

2. Be clearer what humanitarian and development means as related to health beyond the usual phrase "health system strengthening" which is often used without contextualization.

The reviewer’s comment is noted. A more expanded definition of health systems strengthening, drawn from the WHO health systems framework, has now been incorporated into the paper in order to orient the reader on this concept. These additions can be viewed in lines 119-124.

3. The key informants have different backgrounds with varying prejudices and biases. It will be useful to provide a clear analysis of the opinions of the different groups in the results section. This will provide a basis for a more objective analysis to find areas of agreement and divergence, which could be triangulated with the literature to formulate implementable recommendations.

The reviewer’s comment is appreciated. In terms of analysis, a rigorous analysis of key informant perspectives was conducted for this research in line with extensive guidance on qualitative methods for case studies from within the research team and from the literature, including Yin’s *Case Study Research Design and Methods* and Creswell’s *Qualitative Inquiry and Research Design*. Beyond the analysis of interview transcripts in the qualitative software program (Dedoose), we triangulated these findings with the information obtained from different interviewees (especially in instances when these perspectives contradicted or substantiated each other), the document review conducted, and literature. Given this case study was qualitative in nature, the intention of the study was not to validate findings simply by quantifying any of the results or grouping them according to frequency by each type of stakeholder. Rather it was to capture the various challenges, bottlenecks, barriers, and facilitators from the perspectives of humanitarian and development practitioners operating in South Sudan in their implementation of the HDN.

Some of the key recommendations that emerged from the research have been outlined in the discussion and conclusion sections. Examples of those included recommendations for financing, including transition planning between funds and measures to use resources more efficiently (lines 580-592); enhancing coordination measures and engagement with government counterparts at the sub-national level, particularly health technocrats (lines 593-600, lines 610-618); and preparedness measures draw from emergencies which may help humanitarian and development actors invest in health system strengthening (lines 682-698).

4. There is too much focus on operationalizing the nexus focusing the three elements on Governance, financing and Coordination without being strongly supported by the how. The discussions should be stronger supported by evidence, including from the literature. There are many publications in the literature recommending how the nexus could be strengthened. How do they compare to the findings in South Sudan.

The reviewer’s comment is noted. Some of the “how” has been outlined in the discussion section following each point made as it pertains to each thematic area (e.g., financing, governance/leadership, coordination), along with drawing from the wider literature on South Sudan. When examples specific to South Sudan were not available (based on the literature review conducted), other fragile settings were cited. Literature referenced that shows how the nexus may be strengthened, or what challenges continue to prevent this strengthening, have been cited in the discussion. Some of these examples of how to strengthen the nexus (or how to address some of the challenges) can be viewed in lines 580-592; 593-600; 682-700.

5. There are factors missing in the paper which could strengthen the arguments: a) How are the actors (Donors, Humanitarian, and Development) held accountable for all the resources being injected into the system vis a vis their performance. How could this be strengthened? b) Throughout the paper there is no linkage of the findings with the health indicators, except some mention of maternal and child mortality in the introduction session.

The reviewer’s comment is noted. In terms of the first point (a) above, this ties in closely with reviewer comment #4. Some of these recommendations for how to operationalize the nexus more effectively have now been more explicitly incorporated into the manuscript. Examples of these include lines 580-592, 593-618, and 682-700.

In terms of point (b) above, the introduction of the case study provides an overarching health situation overview in South Sudan, including some of the most frequent shocks and stresses (floods, disease outbreaks), weak health system, and some of the poorest health indicators globally (e.g., maternal and child mortality). Beyond this overview to orient the reader, the objective of the paper is to examine the health system/sector at large in South Sudan, including how services are financed, led/governed, and coordinated at-large between humanitarian and development stakeholders. Therefore, the paper focuses on some of these macro-level components of the health sector (e.g., coordination of implementing actors, financing of the health sector, governance structures) and deliberately does not delve into granular detail of any one specific health indicator or outcome given this not within the scope of this research.

6. From 4 and 5 above, the discussions and conclusions should be strengthened by better articulating literature and findings, as well clearly articulating the benefits of doing and consequences of not doing so. This should be supported by the existing data from South Sudan on health indicators.

The reviewer’s comment is noted. As mentioned in comments 4 and 5 above, the discussion and conclusion include some of the key recommendations that can be drawn from the findings of the case study which may help strengthen the implementation of the humanitarian-development nexus for the health sector. Some examples of the lines that include recommendations include lines 580-592, 593-618, and 682-700. Existing data has been used as it pertains to the macro-level components of financing, coordination, and governance/leadership as that was the scope of this research. Granular level detail of health indicators (beyond those referenced in the background section and some of the findings where appropriate) such as specific disease outcomes are not within the scope of this research.

7. A limitation for this study which should be mentioned is non extension of the nexus to the peace element as Humanitarian-Development-Peace. A lot of the factors shaping the landscape in Juba are related to conflict and peace.

The reviewer’s comment is appreciated. The peace component is absolutely critical and relevant to the implementation of the HDN in South Sudan. The topic commissioned for this research was the humanitarian-development nexus and health, not the humanitarian-development-peace nexus. Therefore, an explicit focus on peace was not within the scope of this research. However, we have stated that there are limitations to implementing the HDN if peace has not been realized in South Sudan (lines 282-288). In addition, we have now incorporated a more explicit statement related to the scope of the research in the objectives section (lines 148-151) to make it clear to the reader why peace was not a standalone focus of this research at this stage despite its importance. We did not include this statement in the limitations, as our limitations section only relates to elements of the research that fell within its original scope.

**REVIEWER #2:**

General: The article successfully demonstrated the impact of leadership and governance, financing, and coordination on the operationalization of health interventions in the humanitarian-development nexus in South Sudan.

Thank you for your thoughtful feedback.

1. Although in Leadership and Governance: The limited government investment and engagement, compounded by sanctions and a lack of political will, have perpetuated reliance on international assistance and created barriers to effective coordination and sustainability of health services, article highlight the contributory economic and political shocks that serve as bedrock for this.

Thank you for your thoughtful feedback. We hope that the article clearly articulates this statement.

2. **Also in Financing,** while significant reductions in development health funding have complicated progress towards long-term development objectives, leading to the closure of health facilities and increased reliance on humanitarian funding to fill gaps, article should demonstrate the crowding out effect of humanitarian funding to development funding with data.

The reviewer’s comment is noted and appreciated. The manuscript has now incorporated additional financial data to demonstrate to the reader the shortage of humanitarian funding required to cover both humanitarian needs and the gaps in development assistance between 2022-2023 in order to demonstrate to readers the scale of needs is well beyond assistance available (lines 395-397).

3. **Under Coordination:** Fragmented coordination among humanitarian and development actors, along with structural silos within multi-mandate agencies, has hindered efficient resource use and effective implementation of health interventions. Article should indicate reasons why there is limited political buy-in for the platform

The reviewer’s comment is noted. The reasons for limited political buy-in have been highlighted in lines 654-660, including the nascency of the platform making it too early to assess if stakeholders were able distinguish its success and improvement from the previous version of the HDP coordination platform. In addition, there is the perception that this perpetuated top-down approach and limited localization.

**DATA:**

- Author could consider anonymizing the key informants …KI1; KI2; KI3; KI4 ….Kin.

The reviewer’s comment is noted \. Direct quotes included within the manuscript have now been revised to identify informants as “KI#” rather than “I#” after each quote.

- Consider using number of KI whose opinion coalesced around major theme to strengthen discussion.

The reviewer’s comment is noted. This point was addressed above in response to reviewer #1, comment 3, which will also be included here in response to feedback. Given this case study was qualitative in nature, the intention of the study was not to validate findings simply by quantifying any of the results or grouping them according to frequency by each type of stakeholder, rather it was to capture the various challenges, bottlenecks, barriers, and facilitators from the perspectives of humanitarian and development practitioners operating in South Sudan in their implementation of the HDN.

A rigorous analysis of key informant perspectives was conducted for this research in line with extensive guidance on qualitative methods for case studies from within the research team and from the literature, including Yin’s *Case Study Research Design and Methods* and Creswell’s *Qualitative Inquiry and Research Design*. Therefore, we made the decision to avoid/limit quantifying KI perspectives and relied on other methods to validate results, including triangulation as mentioned above.

**SPECIFIC COMMENTS:**

- Authors to review lines 104 to 107: did those stated characteristics influence choice of study area; are they confounding factors?

The reviewer’s comment is appreciated. These points have been addressed in the limitations section given Juba and Bor were the only study sites selected for this research, thus potentially limiting the replicability of the research and influencing some of the findings that emerged. We emphasize that our selection of these sites was influenced by access constraints in other parts of the country along with financial, logistical, and security reasons. In addition, interviews conducted in Juba capital were intended to represent broader, country-level findings, but we recognize this does not guarantee or signify country-wide phenomena. In addition, we have stated that the findings of this study are not nationally representative since we only collected data in Juba and Bor.

- Authors should clarify line 141 vs 179…. interviews conducted onsite and use of zoom.

The reviewer’s comment is noted. This has now been clarified in the text. A total of 38 KIIs were conducted in-country (KIIs 1-21; KIIs 25-41) and three via Zoom (KIIs 22-25) due to scheduling conflicts (see lines 234-235).

- Review line 498…the fact that there is no clear distinction between humanitarian and development assistance should be a facilitating factor to close the gap in HDN. Development workers should be able to draw synergy at planning to utilize funds to cover issues of development beyond humanitarian assistance.

The reviewer’s comment is noted. Indeed, this introductory sentences of the discussion in lines 554-565 sets the stage for the rest of the points to be made in this section. This includes how complementarity and synergy between humanitarian and development assistance may be enhanced, by drawing from the findings of the study, other examples presented in the discussion drawing from research and implementation examples in South Sudan (e.g., *Jones et al.; Sami et al.*) along with other countries/regions (e.g., the Sahel, Yemen, Afghanistan).
